# Caregiver Qualities and Resident Satisfaction in Long-Term Care: Mediating Roles of Spending Time and Environment

**DOI:** 10.3390/healthcare14070897

**Published:** 2026-03-31

**Authors:** Xiaoli Li, Cheng Yin, Elias Mpofu

**Affiliations:** 1 School of Health Sciences, Southern Illinois University Carbondale, Carbondale, IL 62901, USA; 2 Rehabilitation and Health Services, University of North Texas, Denton, TX 76203, USA; chengyinunt@gmail.com (C.Y.); elias.mpofu@unt.edu (E.M.); 3 School of Health Sciences, University of Sydney, Sydney, NSW 2006, Australia; 4 Department of Educational Psychology, University of Johannesburg, Johannesburg 2006, South Africa

**Keywords:** resident satisfaction, caregiver qualities, spending time, environment

## Abstract

**Background**: Caregiver and resident interactions are important to resident satisfaction with long-term care (LTC). However, these are variously operationalized, and caregiver–resident interactions of “spending time” (activity and autonomy) and environmental quality are less well investigated modifiable factors to inform LTC resident support policies for health aging. **Methods**: This quantitative, cross-sectional study analyzed secondary survey data from 326 long-term care facility (LTCF) residents (aged ≥60) across Shanghai, Nanjing, and Changsha, China. Satisfaction was measured using the Chinese version of the Ohio Long-Term Care Resident Satisfaction Survey. Caregiver Qualities served as the primary predictor, with spending time and environment as parallel mediators. Analysis adjusted for age cohort, functional independence, and length of stay. **Results**: Caregiver qualities were positively associated with overall satisfaction (β = 0.30, *p* < 0.01). Spending time (effect = 0.14, 95% CI: −0.01 to 0.30) and environment quality (effect = 0.05, 95% CI: −0.03 to 0.15) showed non-significant mediated pathways between caregiver qualities and satisfaction, but the combined indirect effect of these domains was statistically significant (effect = 0.19, 95% CI: 0.04 to 0.36). The direct association between caregiver qualities and satisfaction remained significant after accounting for these mediators (effect = 0.36, 95% CI: 0.11 to 0.61). **Conclusions**: These findings clarify how caregiver interactions are important to resident satisfaction both directly and indirectly through spending time, activity engagement, and environmental perceptions. To promote longevity and healthy aging in LTCFs, providers should prioritize caregiver training that fosters resident autonomy, supports daily activity, and maintains age-responsive care environments.

## 1. Introduction

Resident satisfaction is a core indicator of quality in long-term care facilities (LTCFs). International research shows that satisfaction is shaped by care processes, staff–resident interactions, and the physical and social environment [1,2,3]. Beyond clinical outcomes, it reflects how effectively LTCFs support comfort, meaningful daily life, and individualized care [4,5]. As a multidimensional construct, satisfaction encompasses perceptions of care quality, autonomy, interpersonal relationships, engagement, environmental comfort, and overall quality of life [6]. Higher satisfaction is associated with better psychosocial well-being, emotional adjustment, and perceived dignity [7,8]. Resident evaluations are influenced by individual characteristics such as age, functional independence, and length of stay [2,5,9]. Studies in Chinese LTCFs similarly identify these factors as significant predictors of overall satisfaction [10]. Although both personal and organizational determinants have been identified, limited research clarifies how these factors jointly shape overall satisfaction. Addressing this gap is essential for informing person-centered care improvement in LTCFs.

### 1.1. Caregiver Interactions and Resident Satisfaction

Caregiver–resident interactions are among the most consistently identified determinants of resident satisfaction in long-term care settings. High-quality interactions characterized by respect, kindness, attentiveness, and effective communication are strongly associated with higher satisfaction and improved psychosocial outcomes [7]. Frontline caregivers, including nursing assistants, play a particularly influential role due to their extensive involvement in residents’ daily care [11]. A systematic review demonstrated that caregiving staff behaviors and relational care practices are strongly associated with resident satisfaction and perceived care quality across LTC contexts [12]. Organizational factors such as staffing adequacy, training, and supervisory support which further influence caregivers’ ability to provide responsive and person-centered care [13].

### 1.2. Spending Time: Activity, Autonomy, and Engagement Mediation

Opportunities for meaningful activity and engagement are central to quality of life in LTCFs. Engagement in daily, recreational, and social activities has been associated with higher satisfaction, better mood, and improved psychosocial well-being among residents [4,14]. Autonomy in choosing how to spend time is a key element of person-centered care (PCC) and contributes to residents’ sense of control and dignity [15]. Studies conducted in Chinese LTCFs similarly highlight activity opportunities and flexibility in daily routines as important predictors of resident satisfaction [10]. Because caregivers often facilitate or limit residents’ opportunities for engagement, satisfaction with spending time may represent a pathway through which caregiver quality influences overall satisfaction with person centered care.

### 1.3. Environment: Physical and Social Surroundings Mediation

The physical and social environment of LTCFs—including cleanliness, safety, noise levels, spatial layout, and homelike features—plays an important role in shaping residents’ daily experiences. Environmental features that support privacy, autonomy, and social interaction have been associated with higher satisfaction and well-being [16,17]. Person-centered environmental design has been shown to enhance comfort, reduce stress, and support meaningful engagement among older adults [16]. Recent studies in China further demonstrate that environmental quality aligned with PCC principles is a core organizational determinant of resident satisfaction [18].

### 1.4. Theoretical Framework: Person-Centered Care

The PCC framework provides a well-established theoretical foundation for understanding resident satisfaction in LTCFs. PCC emphasizes recognizing residents as individuals with unique histories, preferences, and values, rather than as passive recipients of task-oriented care [15]. Core principles of PCC include dignity, respect, autonomy, meaningful engagement, and supportive relationships—domains closely aligned with satisfaction outcomes in long-term care [6,14].

From a PCC perspective, qualities of caregivers are associated with resident satisfaction both directly and indirectly. A direct satisfaction effect, for example, is that when residents receive care from caregivers who are respectful, attentive, and responsive to resident needs, they are more satisfied. An indirect satisfaction effect is the quality of residents’ daily life within the LTCF. For instance, when residents experience daily living services that are responsive to their preferences for a sense of autonomy in choice activities and spending time meaningfully, they are more satisfied (spending time domain). Similarly, when residents experience the LTCF to be a secure environment that respects their privacy, they also feel safe and comfortable in shared spaces (environment domain). 

McCormack and McCance’s Person-Centered Practice framework further conceptualizes this satisfaction as an outcome of interactions among care processes, caregiver relationships, and the care environment. Therefore, this study examines whether spending time and environment domains are parallel mediators in the relationship between caregiver qualities and overall resident satisfaction.

### 1.5. Study Purpose

**Figure 1:**
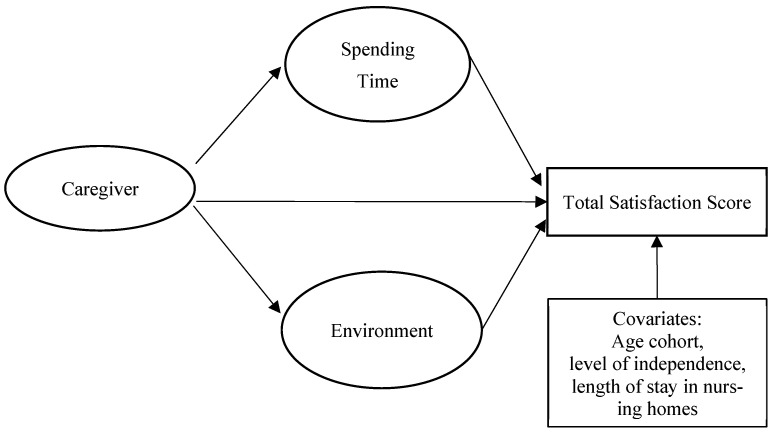
Conceptual framework for this study.

Although prior research, including our earlier analysis using the Chinese version of Ohio Long Term Care Resident Satisfaction Survey (OLCRSS) [18], identified caregivers, spending time, and environment as significant predictors of resident satisfaction in Chinese LTCFs [10], the mechanisms linking these domains remain unclear. The present study applies a parallel mediation model to examine whether satisfaction with spending time and environment mediates the relationship between caregiver qualities and overall resident satisfaction, while adjusting for age cohort, functional independence, and length of stay. The study tested the following hypotheses controlling for age cohort, level of independence, and length of stay in nursing homes (see Figure 1 for conceptual model).

Based on the literature review, we tested the following hypotheses.

**Hypothesis 1.**  *Higher caregiver qualities are associated with higher total satisfaction scores among LTCF residents.*

**Hypothesis 2a.**  *Higher caregiver qualities are indirectly associated with higher total satisfaction through higher satisfaction with spending time.*

**Hypothesis 2b.**  *Higher caregiver qualities are indirectly associated with higher total satisfaction through higher satisfaction with the environment.*

**Hypothesis 2c.**  *Spending time and environment jointly mediate the association between caregiver qualities and total resident satisfaction for higher resident satisfaction.*

## 2. Methods

### 2.1. Study Design

This follow-up study used a quantitative, cross-sectional design based on a secondary analysis of data from a previously completed survey of LTCF residents in China, in which our team collected primary survey data. The original survey administered the OLCRSS in LTCFs located in Shanghai, Nanjing, and Changsha between June and December 2023 and was designed to examine personal and organizational predictors of resident satisfaction.

For the present analysis, we re-used the same resident level dataset to investigate how selected organizational care domains are related to overall satisfaction through potential mediating pathways, while accounting for key personal characteristics. No new data were collected, and all cases were de-identified records from the prior study.

### 2.2. Participants and Setting

Participants were residents of LTCFs located in three large cities in China (Shanghai, Nanjing, and Changsha). Facilities were recruited through existing collaborations with local administrators, and all eligible residents were invited to take part in a paper-based survey during routine facility activities between June and December 2023.

Eligibility criteria for the original survey included being 60 years of age or older, living in the LTCF for at least one month, and being able to speak and read simplified Chinese Mandarin. Residents also needed to be judged cognitively able to understand and answer the questionnaire. Individuals with documented dementia or severe cognitive impairment in their medical records or by caregiver report, those with profound hearing or speech difficulties that prevented effective communication, and residents receiving end-of-life care were not approached for participation.

A total of 399 residents completed the questionnaire. After excluding participants who refused to disclose their information or did not complete the survey (n = 45), the sample size was 354. Missing data were assessed through frequency analyses. Each variable had missing data for 2 to 23 cases (less than 10%), except for the total satisfaction score, which had missing data for 15% of the cases. For variables with less than 10% missing data, mode substitution was applied to dichotomous and ordinal variables, while mean substitution was used for continuous variables.

To determine the nature of the missing data in the total satisfaction score, missing cases were coded as zero, and non-missing cases were coded as one. A binary correlational analysis of the recoded variable along with other variables was conducted, which showed no statistically significant correlations. This indicates that the pattern of missing data is likely to be Missing at Random (MAR) [19]. Therefore, the missing data in this variable was most likely due to participant omissions, and mean substitution was used for this variable [20].

Outliers in the dataset can impact regression results. To address this, Mahalanobis distances and scatter plots were used to detect outliers [21] leading to the identification and removal of 28 outliers. This resulted in a final sample of 326 participants for this study.

The participating LTCFs reflect the contemporary Chinese context in which such facilities serve both highly dependent residents and relatively younger or healthier older adults who choose institutional care for convenience, social engagement, or to reduce caregiving burden on families.

### 2.3. Measures

Outcome variable. Resident satisfaction was measured with the Chinese adaptation of the OLCRSS and was the outcome variable. The OLCRSS measures how residents rate different aspects of care in the facility. Prior testing of this instrument has shown excellent psychometric performance, with a content validity index of 1.0, an intraclass correlation coefficient of 0.96 (*p* < 0.001), and an internal consistency coefficient (Cronbach’s alpha) of 0.96 [18]. For this study, the outcome variable was the total satisfaction score derived from the OLCRSS and measured as a continuous variable ranging from 0 to 100. A higher score shows better total satisfaction.

Construction of weighted domain scores. Domain scores were created using the method of regression-weighted composite scoring as described in our prior analysis of this Chinese OLCRSS resident dataset. Briefly, the previously published analysis created domain scores by fitting linear regressions for each OLCRSS domain using total satisfaction score as the outcome and the items comprising each domain as predictors. The regression coefficients for each item were then used to weigh the items when calculating domain scores. Therefore, those items, more strongly associated with total resident satisfaction had a larger impact on the domain score. Regression-weighted composite scoring was used as it may better reflect each item’s contribution to overall resident satisfaction. As this is a secondary analysis of previously collected data, these weights were carried over and were not re-derived or validated in a separate sample.

Predictor variable. Resident assessment of caregiver qualities, measured by the caregiver’s domain of the Chinese OLCRSS, was the predictor variable. This domain contains items that measure the perceptions of direct care staff, including their kindness, respectfulness, communication skills, attentiveness to care and daily living needs, and dependability. Items in the caregiver’s domain were measured on a 5-point Likert scale (1 = no, 2 = probably no, 3 = neutral, 4 = probably yes, 5 = yes). In a previous study, the reliability of resident assessment scores was 0.89 [18]. For the current analysis, we transformed item scores in the caregiver’s domain to a regression-weighted composite score using the item weights from the linear regression models of total satisfaction on the domain items in our prior study. A higher score on this composite represents a higher level of caregiver satisfaction.

Mediators. The two mediators were spending time and environment. These were rated as follows.

Spending time. The spending time domain represents the extent to which residents have the ability to select and enjoy daily activities, including recreational activities, socializing with others, and flexibility in engaging in routines that are personally meaningful. Items were scored on a 5-point Likert scale as the predictor variable. Item scores within each domain were summed to create a regression-weighted composite score, where the item weights were based on linear regression models of total satisfaction on the domain items based on our previously published paper. A higher composite score represents a higher level of satisfaction on spending time domain. The reliability of spending time scores was 0.83 [18].

Environment. The environment domain reflects perceptions of the physical and social surroundings in the LTCF, such as cleanliness, safety, noise level, comfort of shared and private spaces, and the extent to which the facility feels homelike. Items are rated using the same 5-point Likert scale as above. Item scores within this domain were also combined into a regression-weighted composite score using the same procedure, with higher values representing a more positive evaluation of the facility environment. In a previous study, the reliability of Environment scores was 0.84 [18].

Covariates. Following our previously published study on LTCF resident satisfaction, we included age cohort, level of independence, and length of stay in nursing homes as covariates [22,23]. Age cohort refers to the age range consisting of four levels [60–69 (reference group), 70–79, 80–89, and 90 years or older]. Level of independence was coded with three groups [independent (reference group), half independent, and completely dependent]. Length of stay in the nursing home was categorized into four groups [0–2 months (reference group), 3–12 months, 1–3 years, and more than 3 years]. Figure 1 shows the conceptual framework for this study.

### 2.4. Procedure

The study was approved by the Institutional Review Board of the University of North Texas (IRB-21-250), and all participants provided written informed consent before enrollment. For the present follow-up analysis, only the de-identified dataset from this original survey was used, and no additional contact with residents was required.

### 2.5. Data Analysis

All analyses were performed using the Statistical Package for Social Sciences (SPSS, version 29). Means and standard deviations were reported for continuous variables and frequencies and percentages for categorical variables. Pearson correlation coefficients were used to determine the intercorrelations between the three domains and the total satisfaction score. Linear regression models were used to assess the relationship between each organizational domain score and total satisfaction score adjusting for age cohort, level of independence, and length of stay in the nursing home.

Before interpreting the final model, several regression diagnostics were run to assess assumptions. Tolerance and variance inflation factors were calculated to test for multicollinearity among variables. Tolerance values ranged from 0.695 to 0.982, with VIF values ranging from 1.018 to 1.440, which are not indicative of multicollinearity. Scatterplots of standardized residuals versus standardized predicted values were examined for linearity and homoscedasticity. The assumption of normality was tested with a histogram and normal P-P plot of standardized residuals, and these plots did not reveal any serious violations.

Parallel mediation analysis. The mediation analysis was determined using Model 4 of PROCESS version 4.2 for SPSS, focusing on the continuous total satisfaction score as the outcome, as per the methodology outlined by Hayes [24]. We tested the 95% confidence interval (CI) for mediation effects using 5000 bootstrap samples to determine the theoretical model. If the 95% CI did not encompass 0, then the findings were considered statistically significant, indicating that spending time or environment indirectly influenced the relationship between caregiver domain and total LTCF resident satisfaction.

## 3. Results

### 3.1. Descriptive Statistics

Table 1 summarizes the key demographic and clinical characteristics of the 326 LTCF residents and the mean scores for satisfaction domains. Nearly half of the residents were aged 80–89 years (49.7%), followed by those aged 70–79 (24.5%), 90 years or older (15.3%), and 60–69 (10.4%). About two thirds of the sample were female (66.0%). Regarding functional status, 47.9% were classified as half independent, 39.9% as independent, and 12.3% as completely dependent. More than half of the residents had lived in the nursing home for 1–3 years (51.5%), whereas 31.6% stayed 3–12 months, 9.6% 0–2 months, and 7.4% more than 3 years. The mean total satisfaction score was 89.22 (SD = 10.22). Mean composite scores for the organizational domains were 27.62 (SD = 2.62) for caregivers, 21.77 (SD = 2.75) for spending time, and 27.62 (SD = 2.44) for environment. Detailed demographic and clinical characteristics of the analytic sample are presented in Table 1.

### 3.2. Composite Satisfaction Domain Correlations

The intercorrelations among the predictor, mediators, and outcome variables show that all three composite organizational domains were significantly positively related to total satisfaction. Correlations with total satisfaction were small to moderate in magnitude (Table 2), ranging from r = 0.16 (environment) to r = 0.31 (caregivers).

The strongest association among the domains was observed between caregivers and spending time (r = 0.61), followed by caregivers and environment (r = 0.44) and spending time and environment (r = 0.41).

### 3.3. Associations Between Organizational Domains and Total Satisfaction

Table 3 presents the results on the associations between the three organizational domains and total satisfaction among LTCF residents.

The caregiver’s domain was positively associated with total satisfaction (β = 0.30, *p* < 0.01), and the model including caregivers and the covariates explained 17% of the variance in total satisfaction (R^2^ = 0.17). Spending time also showed a positive association with total satisfaction (β = 0.13, *p* < 0.01), with the corresponding model accounting for 15% of the variance (R^2^ = 0.15). Similarly, the environment domain was positively related to total satisfaction (β = 0.17, *p* < 0.01), and the model including environment and the covariates explained 12% of the variance in total satisfaction (R^2^ = 0.12). Thus, Hypothesis 1 (higher caregiver qualities are associated with higher total satisfaction scores among LTCF residents) was supported.

### 3.4. Mediation Effect of Spending Time and Environment Domains on Total Satisfaction in LTCF Residents

A parallel mediation analysis was conducted to examine the relationship between caregiver qualities and total satisfaction through two mediators of spending time and environment.

**Figure 2:**
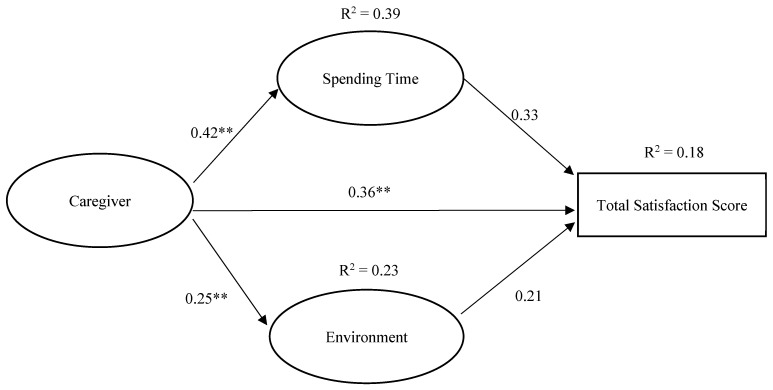
Parallel mediation model of the relationship between caregiver, spending time, environment, and total satisfaction score. ** *p* < 0.01. The model controls for age cohort, level of independence, and length of stay in nursing homes. The model shows that caregiver, along with covariates, explains 39% of the variance in spending time (R^2^ = 0.39) and 23% of the variance in environment (R^2^ = 0.23). The whole model explains 18% of the variance in total satisfaction score (R^2^ = 0.18).

Figure 2 presents the parallel mediation pathways, including standardized coefficients (β) and the proportion of variance explained (R^2^) for spending time, environment, and total satisfaction. Caregivers, together with the covariates, accounted for 39% of the variance in spending time (R^2^ = 0.39) and 23% of the variance in environment (R^2^ = 0.23). The overall mediation model explained 18% of the variance in total satisfaction (R^2^ = 0.18).

Table 4 summarizes the decomposition of the mediation effects, including the total effect, direct effect, total indirect effect, and the specific indirect effects through spending time and environment. The direct effect of caregivers on total satisfaction remained significant after including the mediators in the model (effect = 0.36, 95% CI: 0.11 to 0.61). The total indirect effect was statistically significant (effect = 0.19, 95% CI: 0.04 to 0.36). However, the specific indirect effect through spending time was positive but not statistically significant (effect = 0.14, 95% CI: −0.01 to 0.30), and the indirect effect through environment was also not statistically significant (effect = 0.05, 95% CI: −0.03 to 0.15), as both 95% confidence intervals include zero. The combined indirect effect of spending time and environment was statistically significant, whereas the specific indirect paths through each individual mediator were not statistically significant when considered separately. Thus, Hypothesis 2c (spending time and environment jointly mediate the association between caregiver qualities and total resident satisfaction) was supported. Contrary to expectation, Hypotheses 2a (higher caregiver qualities are indirectly associated with higher total satisfaction through higher satisfaction with spending time) and 2b (higher caregiver qualities are indirectly associated with higher total satisfaction through higher satisfaction with the environment) were not supported.

## 4. Discussion

This study examined the associations between caregiver qualities and overall resident satisfaction in Chinese LTCFs, with particular attention to the mediating roles of spending time and environment. Building on prior work using the Chinese version of the OLCRSS, the findings provide additional insight into how organizational care domains jointly shape residents’ satisfaction, consistent with PCC theory.

### 4.1. Central Role of Caregiver Evaluations in Resident Satisfaction

Residents’ evaluations of caregivers were positively and moderately associated with overall satisfaction, even after adjusting for age cohort, functional independence, and length of stay. This finding aligns with a robust body of international literature identifying caregiver–resident interactions as among the strongest predictors of perceived care quality and satisfaction in long-term care settings [3,7]. Direct care staff are central to residents’ daily experiences, and their behaviors—such as respect, attentiveness, and effective communication—directly influence residents’ perceptions of dignity, security, and emotional well-being [12].

The present findings are also consistent with prior research in Chinese LTCFs showing that caregiver-related domains explain a substantial proportion of variance in resident satisfaction [10]. From a PCC perspective, these results reinforce the view that satisfaction is not solely a function of structural resources but is strongly shaped by relational care processes that recognize and affirm residents as individuals [14,15].

### 4.2. Contributions of Spending Time and Environment

Both spending time and environment were independently associated with overall satisfaction, although their effects were smaller than those of caregiver evaluations. Opportunities for meaningful activity, engagement and autonomy in daily routines have consistently been linked to higher quality of life and greater satisfaction in LTCFs [3,4]. In the Chinese context, recent studies similarly emphasize that activity choice, flexibility, and opportunities for social participation are important determinants of residents’ psychosocial well-being and satisfaction [8].

Environmental quality—encompassing cleanliness, safety, comfort, and homelike features—has also been widely recognized as a core component of person-centered long-term care environments [16,17]. Although environmental characteristics are often considered relatively fixed or structural, residents’ perceptions of the environment may be shaped by how caregivers support privacy, mobility, and use of shared spaces. This may help explain the observed correlations between environmental satisfaction and caregiver evaluations in the present study.

The mediation analysis showed that the combined indirect effect of spending time and environment significantly mediated the relationship between caregiver evaluations and overall satisfaction, whereas the specific indirect effects of each mediator alone were not statistically significant. This pattern suggests that spending time and environment function as interconnected organizational pathways rather than as independent mechanisms.

Similar conclusions have been reported in prior PCC-oriented research, which emphasizes that care processes, engagement opportunities, and environmental conditions operate synergistically to influence resident outcomes [7,14]. The moderate correlations observed between spending time and environment further support this interpretation and suggest shared variance that may attenuate the detection of distinct indirect effects when mediators are examined separately.

Importantly, the direct effect of caregiver evaluations on satisfaction remained significant after accounting for both mediators. This finding indicates that caregiver–resident interactions influence satisfaction not only through organizational domains such as activity engagement and environment, but also through direct relational mechanisms—such as emotional support, trust, and perceived respect—that are central to PCC but not fully captured by structural or activity-based measures [15].

### 4.3. Implications for Practice and Policy

The findings suggest several implications for practice and policy. First, initiatives aimed at improving resident satisfaction should prioritize caregiver workforce development, particularly training that emphasizes communication skills, respect, and responsiveness—core elements of PCC [7]. Second, activity programming and environmental improvements should be implemented in coordination with caregiver practices, as their combined influence appears more consequential than isolated changes. At the policy level, quality assessment and improvement frameworks may benefit from explicitly recognizing the interdependence of relational care, engagement opportunities, and environmental support in shaping resident satisfaction.

### 4.4. Strengths, Limitations, and Future Research Directions

Strengths of this study include the use of a validated satisfaction instrument, data collected from multiple urban regions in China, and a theoretically grounded parallel mediation approach. Findings would be robust or replicable similar regions of China.

For limitations, the two mediators of spending time nor environment appeared to be overlapping rather than separate in effect which may have attenuated the ability to detect distinct effects for each mediator, particularly if the items are closely related or measured with limited sensitivity. These associations may require further examination considering both mediators that were theoretically and empirically related. Third, the sample size may have reduced statistical power to detect smaller, individual indirect effects, even when the combined pathway reached significance. Therefore, future work with larger samples, longitudinal designs, and intervention testing is needed to demonstrate these pathway-specific effects and test multi-component PCC strategies that concurrently address caregiver practices, activity engagement, and environmental design.

## 5. Conclusions

This study provides evidence that caregiver evaluations are a central determinant of resident satisfaction in Chinese long-term care facilities and that their influence operates both directly and indirectly through organizational domains related to spending time and environment. Although spending time and environment did not independently mediate the caregiver–satisfaction relationship, their combined indirect effect highlights the interconnected nature of person-centered care processes. These findings underscore the importance of integrated care strategies that support caregiver–resident relationships, meaningful engagement, and supportive environments. As China’s long-term care system continues to expand, adopting person-centered approaches that address these inter-related domains may be critical for enhancing resident satisfaction and overall quality of care.

## Figures and Tables

**Table 1:** Demographic and clinical characteristics of residents (n = 326).

Variable	n	%
**Age cohort**		
60–69	34	10.4
70–79	80	24.5
80–89	162	49.7
90+	50	15.3
**Gender**		
Male	111	34
Female	215	66
**Education level**		
Primary school	166	50.9
Middle school	101	31
High school	52	16
Bachelor’s degree or more	7	2.1
**Marriage status**		
Married	155	47.5
Single	52	16
Divorce	23	7.1
Widowed	96	29.4
**Living arrangement**		
Separate room	41	12.6
Twin room	162	49.7
Multiple bedded room	123	37.7
**Level of independence**		
Independent	130	39.9
Half independent	156	47.9
Completely dependent	40	12.3
**Chronic diseases count**		
1	96	29.4
2	164	50.3
3 or more	66	20.2
**Daily activity participation**		
1	108	33.1
2	169	51.8
3 or more	49	15
**Monthly expenditures**		
0–999	7	2.1
1000–2999	123	37.7
3000–4999	94	28.8
5000+	102	31.3
**Length of stay in nursing homes**		
0–2 months	31	9.6
3–12 months	103	31.6
1–3 years	168	51.5
3 years or more	24	7.4
	Mean	Standard deviation
Total satisfaction score	89.22	10.22
Caregivers	27.62	2.62
Spending time	21.77	2.75
Environment	27.62	2.44

**Table 2:** Intercorrelations of predictor, mediators, and outcome variables.

Variable	1	2	3	4
1 Total satisfaction score	1.00	-	-	-
2 Caregivers	0.31 **	1.00	-	-
3 Spending time	0.28 **	0.61 **	1.00	
4 Environment	0.16 **	0.44 **	0.41 **	1.00

** *p* < 0.01.

**Table 3:** Separate adjusted linear regression models for total satisfaction among LTCF residents (n = 326).

Variables	B	SE	β	R^2^	95% CI
Caregiver ^a^	0.94	0.16	0.30 **	0.17	0.63–1.26
Spending time ^a^	1.38	0.56	0.13 **	0.15	0.28–2.48
Environment ^a^	1.82	0.57	0.17 **	0.12	0.70–2.94

B = unstandardized beta weight; SE = standard error; β = standardized beta weight; R^2^ = total explained variance of total satisfaction score; 95% CI = 95% confidence interval; ** *p* < 0.01; ^a^ separated model for each determinant with covariates of age cohort, level of independence, and length of stay in nursing homes.

**Table 4:** The effects on parallel mediation models on total satisfaction score.

	Effect	Boot SE	Boot LLCI	Boot ULCI
Total effect	**0.55**	0.10	0.35	0.74
Direct effect	**0.36**	0.13	0.11	0.61
Indirect effect				
Total	**0.19**	0.08	0.04	0.36
Spending time	0.14	0.08	−0.01	0.30
Environment	0.05	0.05	−0.03	0.15

CI = confidence interval; SE = standard error; LL = lower limit; UL = upper limit; All included covariates of age cohort, level of independence, and length of stay in nursing homes; Bolded values indicate statistically significant results.

## Data Availability

The original contributions presented in this study are included in the article. Further inquiries can be directed to the corresponding author.
